# Experimental dataset on preparation and characterization of black sand mineral-based as photocatalyst

**DOI:** 10.1016/j.dib.2020.105373

**Published:** 2020-03-10

**Authors:** A. López-Vásquez, J.A. Colina, Fiderman Machuca-Martínez

**Affiliations:** a Department of Chemical Engineering, Facultad de Ingeniería y Arquitectura, Universidad Nacional de Colombia, Campus La Nubia, Manizales 170003, Colombia; b Chemical Engineering Program, Universidad de Cartagena, Av. El Consulado 48-152, Cartagena A.A. 130001, Colombia; c Escuela de Ingeniería Química, Universidad del Valle, A.A. 23360 Cali, Colombia

**Keywords:** Black sand, Geocatalysis, Mineral-based photocatalyst, Natural composite

## Abstract

Heterogeneous photocatalysis with natural minerals (geocatalysis) has been considered for a wide variety of redox processes, which require the participation of metals either as catalysts or promoters in the reactive system. Black mineral sand as raw material (RM), which is a natural composite frequently found in coastal deposits, was used for the preparation of a semiconductor potential. Due to iron content of mineral, it was applied several magnetic fields (0.0311 and 0.1645 Tesla) to obtain the fractions M1 and M2, respectively. The fraction that was not magnetically attracted was named NM. The chemical, structural and optical properties of each obtained composite were characterized by Zeta potential (ζ), X-ray fluorescence (XRF), energy-dispersive X-Ray analysis (EDX), scanning electron microscopy (SEM), BET area, thermal gravimetric analysis (TGA), X-ray diffraction (XRD) patterns, Fourier transform infrared (FT-IR) and UV–Vis spectroscopy techniques.

**Specifications table**

**Table:** 

Subject	Chemical engineering
Specific subject area	Synthesis and characterization of materials
Type of data	Figure and table
How data were acquired	Data were obtained by characterization techniques such as Zeta potential (ζ), XRF, SEM/EDX, TGA, XRD and FT-IR. The single-point BET surface area was determined using the dynamic flow method by nitrogen adsorption. From UV–Vis spectroscopy, Tauc plots were used to estimate the material band-gap energy (E*_g_*),
Data format	Raw and analyzed
Parameters for data collection	Effect of the composition of each fraction obtained by magnetic separation on morphologic, structural and optical properties as photocatalyst.
Description of data collection	The black mineral sand (RM) was collected from coastal deposits (11°10′57.3″N 74°14′18.0″W) taking representative sub-samples from different points. The composite mixture was prepared by quartering method (ASTM D75). This sample was scrubbed and deslimed and taking advantage of its iron content, was separated in fractions applying several magnetic fields (0.0311 and 0.1645 T, respectively), which could be used as semiconductor in photocatalytic processes.
Data source location	Universidad Nacional de Colombia sede Manizales
	City/Town/Region: Manizales, Caldas
	Country: Colombia
Data accessibility	With the article
	Repository: Mendeley Data https://data.mendeley.com/datasets/k7yt9hm7zw/2

## Value of the Data

• Data obtained allow knowing the morphological, structural and optical properties of a mineral-based semiconductor, which could be used as catalyst in photocatalytic processes.
• According their composition and optical response (E*_g_*), the electron transfer mechanism during photocatalytic process could be explained by Z-scheme in a photo-Fenton process.
• The band gap data for each fraction, could serve as reference to use another mineral like semiconductor doped naturally.

## 1 Data

The black sand is a natural mineral that is presented in coastal and adjacent areas to riverbeds, which is constituted by iron titanates (ilmenite) and minor oxides such as calcium, magnesium and silicon [1]. These compounds, pure or coupled, traditionally have been used as semiconductor in numerous photocatalytic processes [2], [3], [4], [5]. The dataset describes the chemical, structural and optical characterization of several fractions obtained magnetically, from this natural composite. The raw and analyzed data files were deposited at Mendeley database (DOI: 10.17632/k7yt9hm7zw.2).

## 2 Experimental design, materials, and methods

### 2.1 Black sand mineral-based photocatalyst preparation

**Fig. 1:**
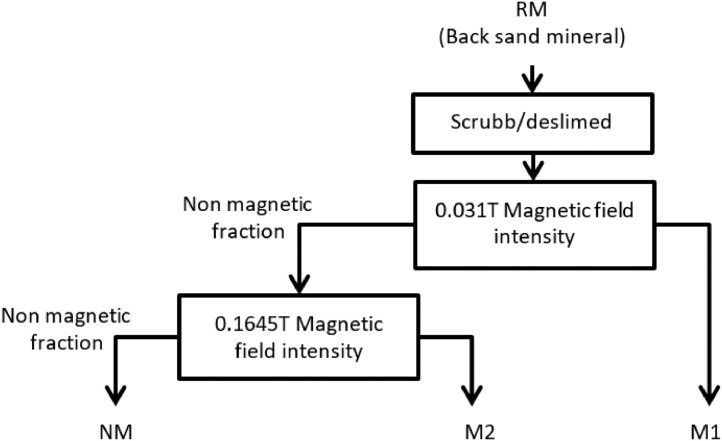
Schematic representation of the sequential processing of the black mineral sand for obtaining black sand mineral-based photocatalyst.

To prepare the photocatalyst from mineral, black sand (RM) samples were collected from coastal deposits taking representative sub-samples from different points. The composite mixture was prepared by quartering method (ASTM D75). The fractions obtained by magnetic separation from raw material, (Fig. 1), were characterized using chemical, structural and optical techniques.

### 2.2 Black mineral sand characterization

The Zeta potential (ζ) of the fractions was established by Zero point of charge (pH_zpc_) determination. For this purpose, a suspension of each fraction was prepared by adding 50 mg of sample (ground under 53 µm) to 100 ml of distilled and deionized water, then, the pH was adjusted using either NaOH or HCl solutions (1 M) over the pH range of 1.5–11.0. The isoelectric point (IEP) was measured as a function of pH by reading the intercept of the trend line with the horizontal line of zero mV (Table 1).

**Table 1:** Isoelectric point of black sand mineral samples as a function of pH.

Sample	Isoelectric point (IEP), pH
RM	2.78
M1	2.47
M2	2.10
NM	2.39

In order to identify the mass distribution and chemical composition of each obtained fraction, fluorescence spectroscopy X-ray (XRF) and energy-dispersive spectroscopic (EDS) analyses were performed. The data are showed in Tables 2 and 3.

**Table 2:** Chemical composition (% wt.) of the fractions obtained from the mineral. Adapted from [6].

Compound and/or element		RM	M1	M2	NM
Major elements	Fe_2_O_3_	75.240	86.819	50.313	13.145
	TiO_2_	14.404	4.548	37.027	10.915
	SiO_2_	4.481	2.608	4.731	45.328
	ZrO_2_	1.245	0.211	0.561	5.335
	MnO	1.010	0.418	2.531	0.741
	Al_2_O_3_	0.941	1.041	1.399	7.566
Trace elements*	CaO	0.829	0.742	1.015	8.203
	Na_2_O	0.462	1.162	0.491	4.069
	MgO	0.411	0.470	0.704	1.708
	Cl	0.304	1.139	0.548	0.779
	P_2_O_5_	0.227	0.430	0.165	0.398
	V	0.175	0.188	0.281	0.094
	Cr	0.121	0.059	N.D	0.079
	K_2_O	0.067	0.086	0.094	0.953
	Nb_2_O_5_	0.032	N.D	0.073	N.D
	S	0.028	0.064	0.031	0.046
	Zn	0.024	0.016	0.037	N.D
	HfO_2_	N.D	N.D	N.D	0.643
	Ce				0.211
	La				0.093
	ThO_2_				0.037
	Y				0.033
	Sr				0.042

⁎ Trace elements < 2.0% w.t.; N.D: Non detectable

**Table 3:** Elemental composition based on EDS analysis of fractions obtained from black mineral sand (wt. %).

Element	Raw material	M1	M2	NM
Fe	46.14	60.33	31.32	3.9
O	34.03	31.27	37.77	48.4
Ti	10.85	1.07	25.46	5.29
Na	4.25	2.10	1.06	6.08
Cl	1.25	–	–	0.67
Al	1.06	1.33	1.12	2.57
Mn	0.98	–	1.73	–
Si	0.75	2.82	0.75	11.13
Mg	0.69	1.09	0.79	0.72
Zr	–	–	–	15.08
Ca	–	–	–	6.15

**Fig. 2:**
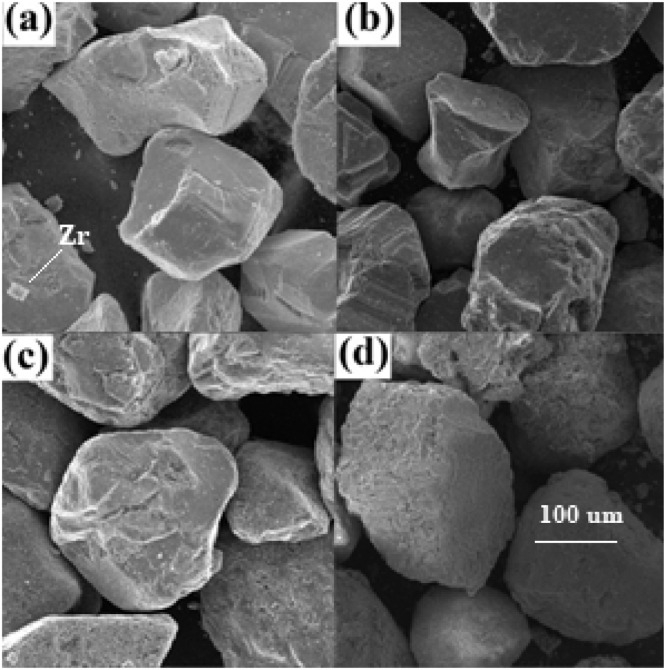
SEM micrographs of black sand and its fractions obtained by magnetic separation: (a) Raw material (Zr crystal incrusted on surface grain), (b) M1, (c) M2 and (d) NM. Adapted from [6].

The Fig. 2 shows the surface morphology of the samples determined by scanning electron microscopy (SEM), while Table 4 shows the surface area of samples (BET) determined by dynamic flow method with nitrogen adsorption at 77.35 K.

**Table 4:** BET surface area of the fractions obtained from black mineral sand.

Sample	BET specific surface area, m^2^ g^-1^
RM	0.5083
M1	0.4928
M2	0.4932
NM	0.5017

**Fig. 3:**
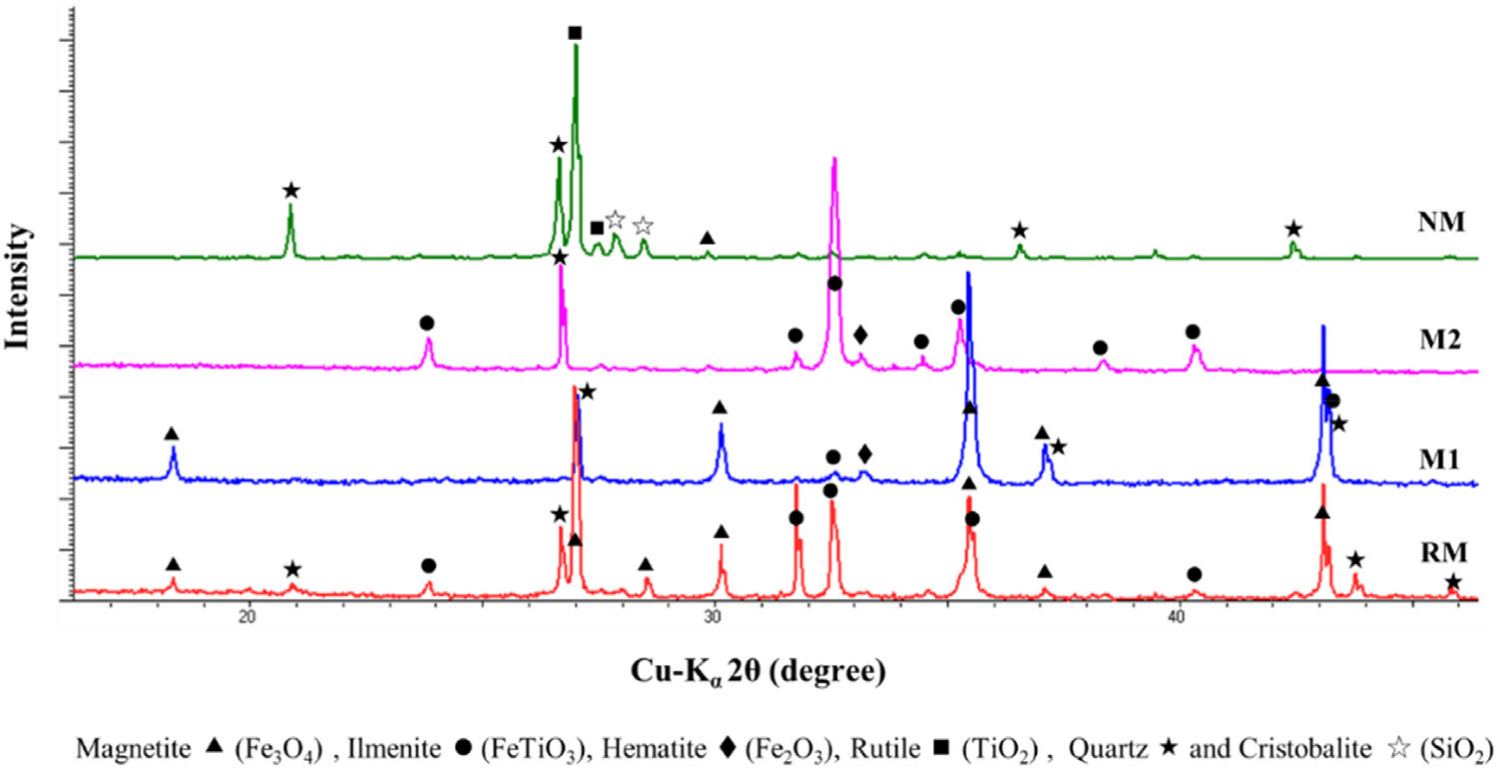
XRD patterns of fractions obtained from the black mineral sand. Raw material (RM), magnetic concentrates (M1 and M2) and non-magnetic fraction (NM). Adapted from [6].

XRD analysis was performed on fractions (Fig. 3) because the samples were composed principally by iron and titanium oxides, which present several crystalline phases, which are important during photocatalytic activity of semiconductor.

**Fig. 4:**
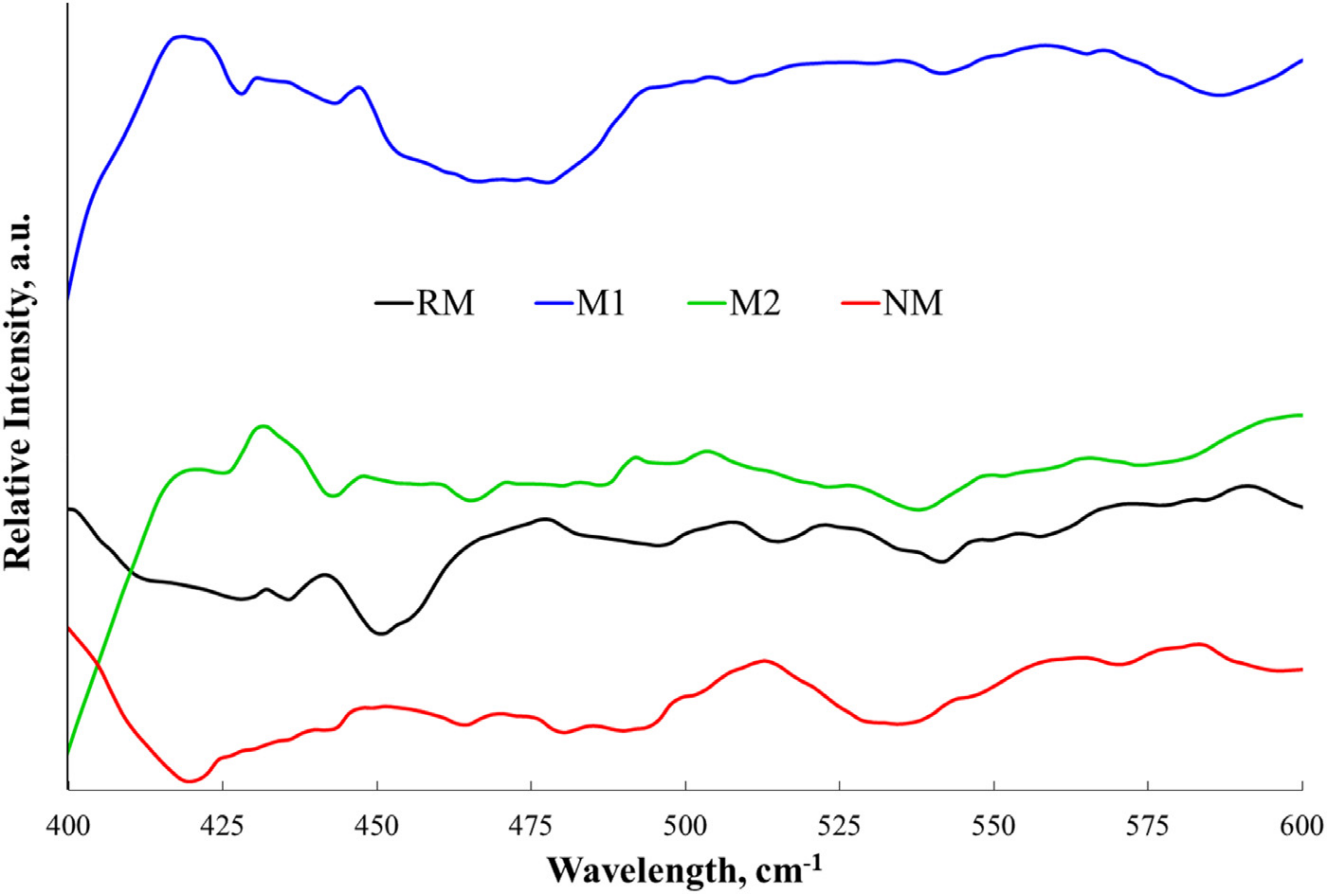
FTIR spectra for fractions obtained from black mineral sand. Adapted from [6].

To identify the formation of alloys among the main components of the mineral, functional groups present were confirmed by Fourier-transform infrared spectroscopy. Fig. 4 describes the comparative assessment of the vibrations within the range 600–400 cm^-1^. In the repository Mendeley Data, the complete raw data can be seen.

**Fig. 5:**
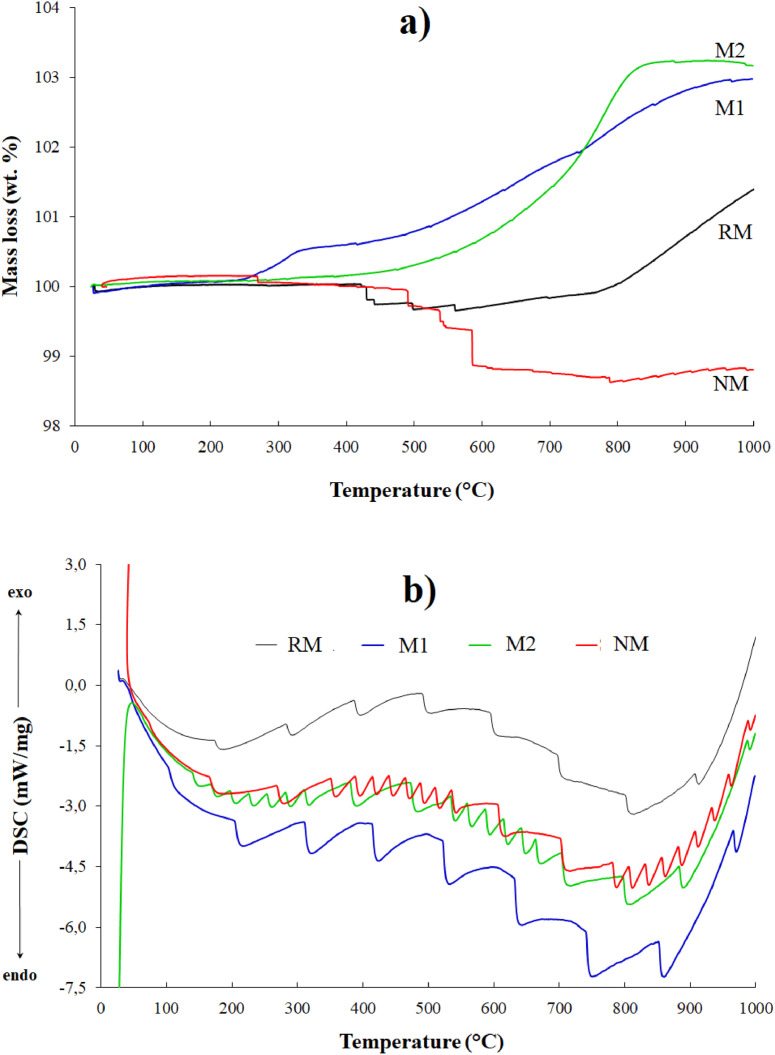
(a) TGA and (b) DSC curves of fractions obtained from black mineral sand. Adapted from [6].

**Fig. 6:**
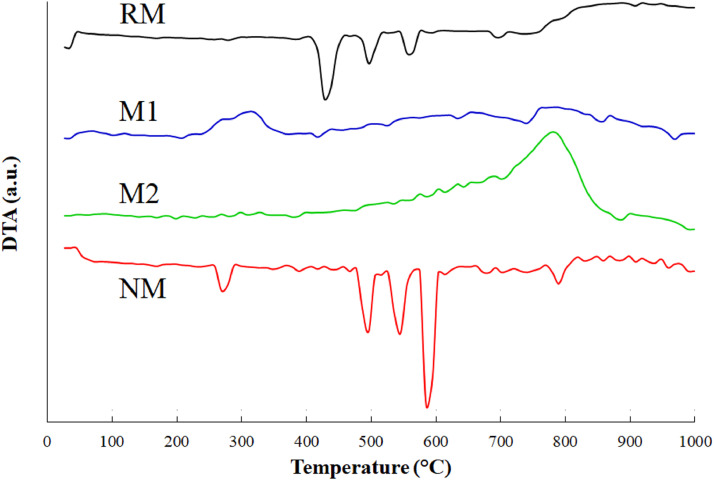
DTA curves for fractions obtained from black mineral sand. Adapted from [6].

Thermogravimetric analysis (TGA/DSC) of the samples was performed to identify the material thermal stability against possible crystalline phase transition (Fig. 5). The endothermic and exothermic transitions as a function of temperature were determined by differential thermal analysis (DTA) (Fig. 6). For these purpose, the samples were degasified at 100  °C during 60 min at 20 cm^3^ min^-1^ in nitrogen purge stream and the analysis were performed since 25  °C to 1000  °C at a heating rate of 5  °C min^-1^ under 100 cm^3^ min^-1^ in flowing air. In the repository Mendeley Data, the complete raw data can be seen.

**Fig. 7:**
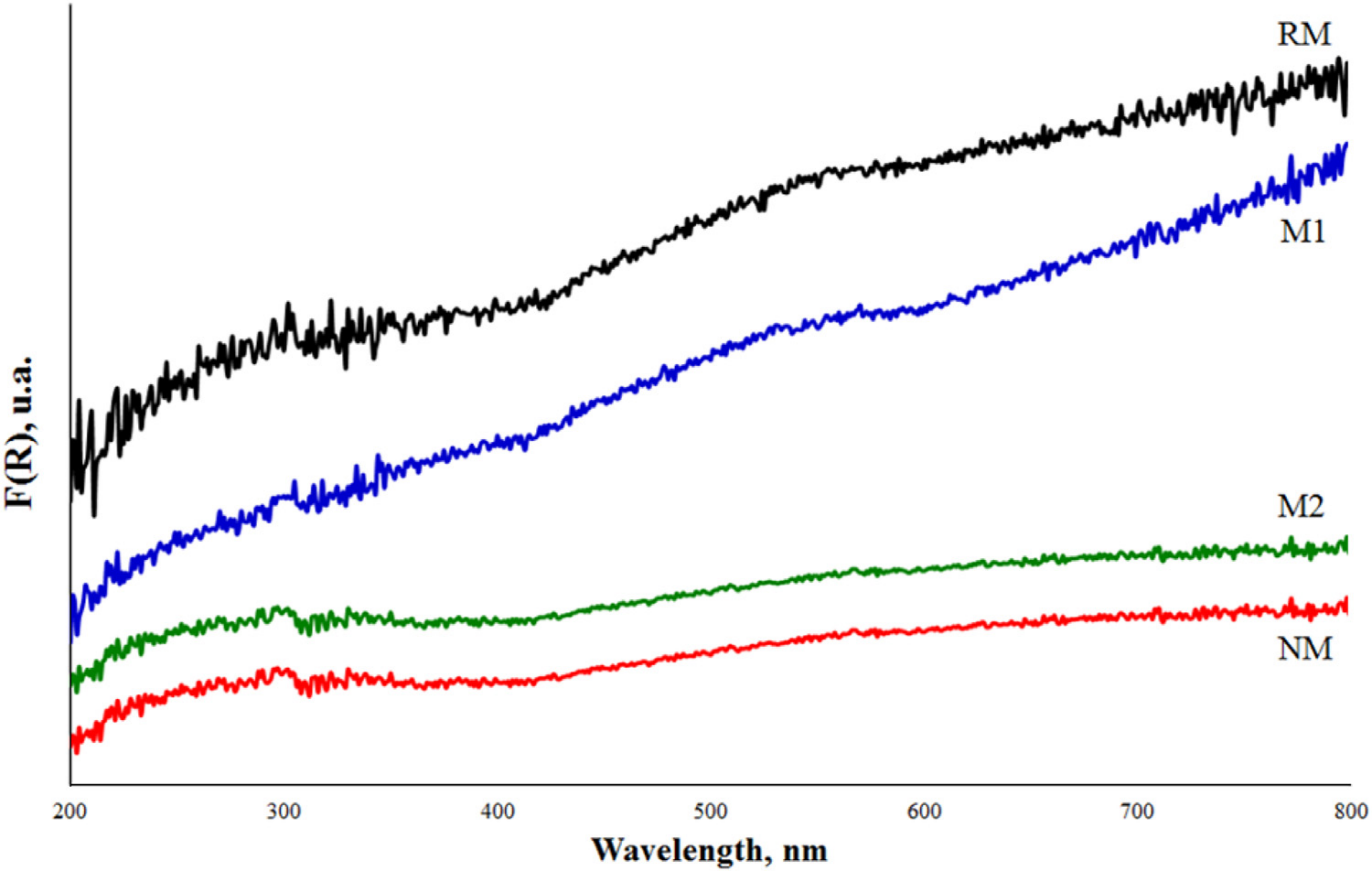
Experimental Kubelka-Munk diffuse reflectance for fractions obtained from black mineral sand.

**Fig. 8:**
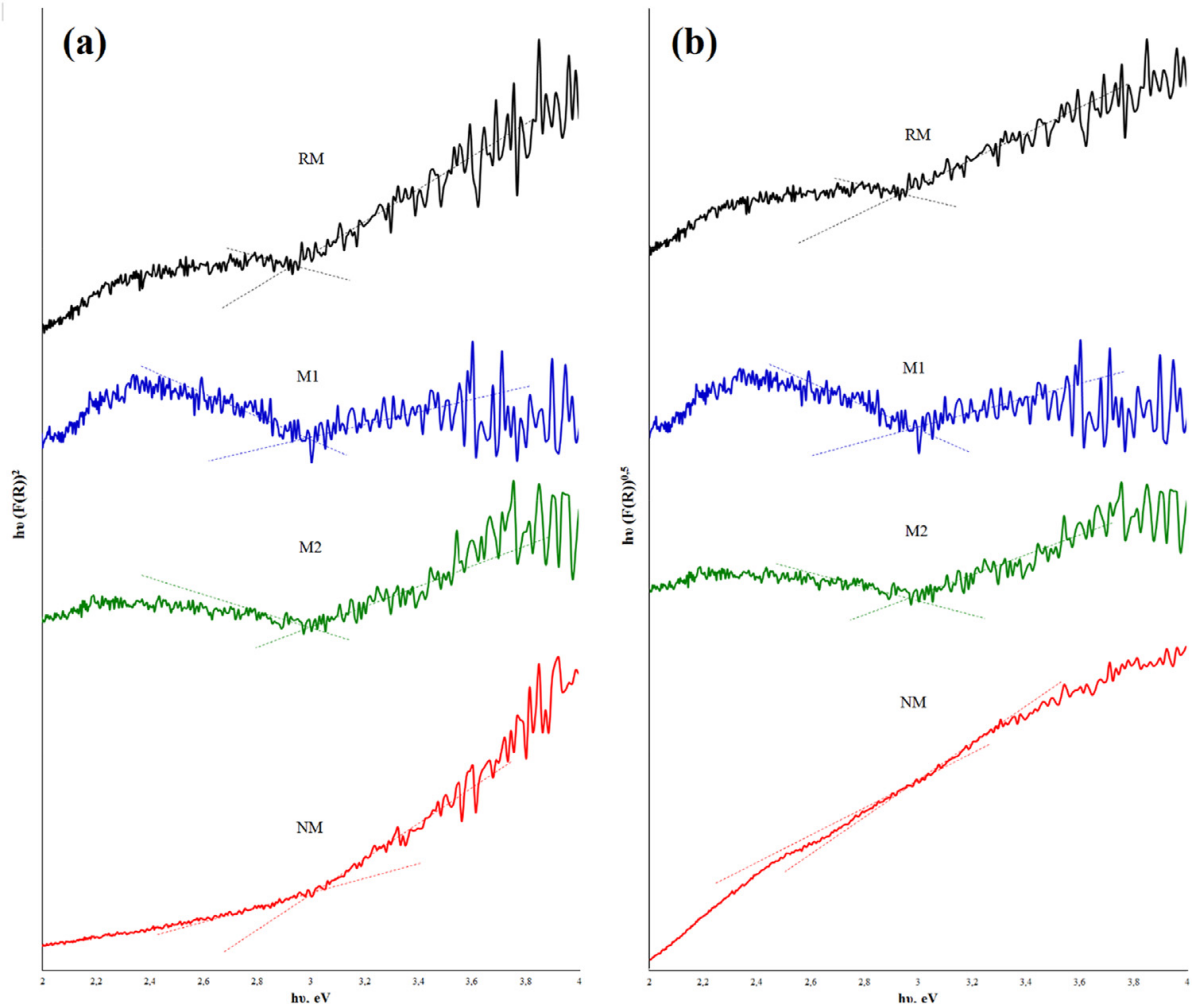
Experimental *E_g_* values obtained from Tauc plot for fractions obtained from black mineral sand. (a) Direct transition, (b) Indirect transition.

The optical response of mineral was determined by the UV reflectance of the solid. For this, the Kubelka-Munk function value for the fractions was determined (Fig. 7) while the Tauc plots were used to estimate the material band-gap energy, E*_g_* (Fig. 8). The direct (n=1/2) and indirect (n=2) optical transitions of the samples [7], are described in Table 5. In the repository Mendeley Data, the complete raw data can be seen.

**Table 5:** Calculated values of band gap for mineral samples.

Band gap, eV	RM	M1	M2	NM
Direct	0.89	0.19	3.00	3.00
Indirect	0.87	0.17	2.98	2.98

## Conflict of Interest

The authors declare that they have no known competing financial interests or personal relationships that could have appeared to influence the work reported in this paper.
